# Body Weight Reduction by Bariatric Surgery Reduces the Plasma Levels of the Novel Orexigenic Gut Hormone Insulin-like Peptide 5 in Patients with Severe Obesity

**DOI:** 10.3390/jcm12113752

**Published:** 2023-05-29

**Authors:** Angelo Di Vincenzo, Marika Crescenzi, Marnie Granzotto, Sara Zancaner, Roberto Fabris, Mirto Foletto, Luca Prevedello, Federico Capone, Roberto Vettor, Marco Rossato

**Affiliations:** 1 Department of Medicine—DIMED, University Hospital of Padova, 35128 Padova, Italy; angelo.divincenzo@unipd.it (A.D.V.); marikacrescenzi@hotmail.com (M.C.); roberto.fabris@aopd.veneto.it (R.F.); roberto.vettor@unipd.it (R.V.); 2 Department of Surgical, Oncological and Gastroenterological Sciences—DISCOG, University Hospital of Padova, 35128 Padova, Italy; mirto.foletto@unipd.it (M.F.);

**Keywords:** obesity, adipose tissue, INSL5, leptin, BMI, bariatric surgery, sleeve gastrectomy

## Abstract

Insulin-like factor 5 (INSL5), a novel hormone secreted by the enteroendocrine cells of the distal colon, has been implicated in appetite and body weight regulation in animals given its orexigenic properties. We investigated basal INSL5 plasma levels in a group of morbidly obese subjects before and after laparoscopic sleeve gastrectomy. Furthermore, we analyzed the expression of *INSL5* in human adipose tissue. Before bariatric surgery, obese subjects showed basal INSL5 plasma levels that were positively correlated with BMI, fat mass, and leptin plasma levels. After weight loss by laparoscopic sleeve gastrectomy, INSL5 plasma levels in obese subjects were significantly lower than those observed before surgery. Finally, we did not detect any expression of the *INSL5* gene in human adipose tissue, both at the mRNA and protein levels. The present data show that subjects with obesity have INSL5 plasma levels positively correlating with adiposity markers. After bariatric surgery, INSL5 plasma levels decreased significantly, and this decrease was not directly due to the loss of adipose tissue since this tissue does not express *INSL5*. Considering the orexigenic properties of INSL5, the reduction of its plasma levels after bariatric surgery in obese subjects could participate in the still unclear mechanisms leading to appetite reduction that characterize bariatric surgery procedures.

## 1. Introduction

Obesity is a chronic disease with a continuously increasing prevalence [1], with important systemic complications including heart disease, diabetes mellitus, hypertension, dyslipidemia, stroke, atherosclerosis, and specific types of cancer [2]. Since obesity is due mainly to an unbalance between energy intake and expenditure, the first-line interventions for overweight and obese subjects are diet and physical exercise that promote calorie restriction and increase energy expenditure. Although these interventions often result in initial weight reduction, the majority of patients with obesity fail to maintain the weight loss in the long term, probably due to many different compensatory changes, above all in appetite regulatory mechanisms [3,4]. On the contrary, bariatric surgery causes substantial long-term weight loss and is an effective intervention for the morbidly obese to achieve marked and long-term weight loss and improve obesity-related comorbidities. For these reasons, bariatric surgery is now considered the most successful method for treating morbid obesity and its associated diseases, such as type 2 diabetes [5].

Bariatric surgery has now quite well-defined beneficial effects on hunger, not only due to gastric restriction but also to the modulation of appetite-regulatory hormones coming mainly from the gut, such as ghrelin, PYY, CKK, and GLP-1 [6,7].

Insulin-like factor 5 (INSL5) is a novel hormone secreted primarily by the enteroendocrine cells of the colon and rectum that has been implicated in both mealtime hunger and the regulation of body weight in animals given its orexigenic properties [8,9,10]. INSL5 belongs to the relaxin/insulin superfamily of peptides consisting of insulin, insulin-like growth factors 1 and 2 (IGF–1 and IGF–2), relaxins 1 and 2, and insulin-like peptides 3, 4, 5, 6, and 7 (INSL 3–7) [11]. Relaxins have many different roles, such as regulation of female and male reproductive tract functions, signaling in the central nervous system, vasodilation and heart stimulation in the cardiovascular system, regulation of fibrotic processes, and wound healing [12].

INSL5, one of the latest identified members of the relaxin superfamily, has been shown to be expressed mainly in the terminal part of the gastrointestinal tract, particularly in the colon and rectum, but it is also expressed in many other tissues as well, including the brain, pituitary, thyroid, kidney, and uterus [13].

Although they are structurally related to insulin, the relaxin family peptides produce their physiological effects by activating a group of four G protein-coupled receptors (GPCRs), named relaxin family peptide receptors 1–4 (RXFP1–4) [11,12].

While it is clear that the relaxin family of peptides has important physiological roles, there are still many unanswered questions about the precise roles of many of them.

In the present study, we evaluated INSL5 plasma levels in a group of patients with obesity before and one year after significant weight and adipose tissue loss obtained by laparoscopic sleeve gastrectomy. Furthermore we investigated the relationship of INSL5 plasma levels with classic obesity related parameters such as BMI, waist circumference, fat mass and with leptin plasma levels. Finally we explored the hypothesis that adipose tissue can be the source of INSL5 production.

## 2. Materials and Methods

### 2.1. Patients

We retrieved stored blood samples from forty morbidly obese patients (29 females and 11 males) previously recruited from the Bariatric Unit of the University Hospital of Padova. Each patient was evaluated before and 12 months after laparoscopic sleeve gastrectomy (LSG) with general clinical parameters (body temperature, blood pressure, heart rate, breath frequency, blood oxygen saturation) and with anthropometric measurements (weight, height, BMI, waist circumference). In all 40 patients, we evaluated glucose and insulin plasma levels together with leptin and INSL5 plasma levels. Blood samples were drawn in the morning (between 8 and 9 a.m.) after an overnight fast. Before and after one year of LSG, twenty-nine out of the forty patients with obesity who underwent bariatric surgery also performed body composition analysis using bioimpedenzometry, as previously described [14].

In our Center LSG is performed as the first-choice bariatric surgery procedure. All patients were operated by the same bariatric surgery team when indicated, according with the NIH consensus criteria for bariatric surgery (BMI higher than 35 kg/m^2^ in the presence of co-morbidities or with a BMI higher than 40 kg/m^2^) [14]. Absolute exclusion criteria included alcohol addiction and severe psychiatric disorders. Full details of the surgical technique have been published recently by our group [15].

### 2.2. Measurement of INSL5 Plasma Levels

Blood samples have been collected into vacutainer tubes containing EDTA. After gentle rocking of the tubes several times immediately after the collection of blood for anti-coagulation, the blood is then transferred from the lavender vacutainer tubes to centrifuge tubes containing aprotinin (0.6 TIU/mL of blood) and gently rocked several times to inhibit the activity of proteinases. Tubes were then centrifuged at 1600× *g* for 15 min at 4 °C, and plasma was collected and kept at −80 °C until INSL5 determination using a commercial EIA kit from Phoenix Pharmaceuticals (#EK-035-70, Burlingame, CA, USA) according to the manufacturer’s instructions. The minimum detection level was 80 pg/mL. The cross-reactivity of the antibody raised against human INSL5 was 0% to human insulin, INSL3, INSL4, INSL6, INSL7 (Relaxin 3), and Relaxin 2. Intra- and inter-assay variations were <10% and <15%, respectively.

Leptin was measured in the same blood samples utilizing an ELISA kit (#E04649H, Cusabio, Houston, TX, USA) with a 0.156 ng/mL–10 ng/mL detection range and a sensitivity of 0.060 ng/mL.

### 2.3. Isolation of Human Adipose Tissue

Subcutaneous adipose tissue (SAT) was obtained from nine subjects undergoing elective surgery for minor abdominal diseases who were otherwise healthy and not taking any drugs. Each SAT sample was processed as previously described [15]. Briefly, all adipose tissue samples were collected during laparoscopic surgery in the abdominal region. In particular, 1 cm^3^ SAT was obtained excising subcutaneous fat at trocar site and then it was gently rinsed in PBS buffer, immediately frozen in liquid nitrogen and stored at −80° C for further analyses.

### 2.4. RNA Isolation and Reverse Transcription PCR (RT-PCR) in Adipose Tissue

RNA isolation from SAT was performed using an affinity column-based method (RNEasy Kit, Qiagen GmbH, Hilden, Germany). After homogenization of 100 mg of tissue in QIAzol Lysis Reagent with TissueLyser, chloroform was added and mixed by vortexing for subsequent phase separation through centrifugation. In the upper aqueous phase, the RNA was added to 1 volume of 70% ethanol and mixed thoroughly by inverting the tube up and down several times. Each sample was then loaded on the RNEasy spin column, and from this step on, the manufacturer’s protocol was followed. At the end, the RNA was eluted in 50 μL of RNAse-free water. Quantity and quality of the RNA were evaluated by a DeNovix DS-11 spectrophotometer (Resnova, Ariccia, Italy) using OD 260 for calculation of the concentration and the ratios 260/280 and 260/230 for assessing the purity of the samples. First-strand cDNAs were accomplished in a 60 min incubation at 37 °C using 2 µg RNA, 200 U/µL M-MLV reverse transcriptase, and 0.5 µg/µL random primers (Promega Corp., Madison, WI, USA). A quantity of 10 ng of cDNA was used to detect Insulin-like 5 (INSL5) gene expression on the Veriti 96-Well Thermal Cycler (ABI Applied Biosystems, Waltham, MA, USA) using Pfu DNA Polymerase (Promega Corp., Madison, WI, USA). The ribosomal protein lateral stalk subunit P0 (RPLP0) housekeeping gene was used to avoid deviations from the process of measurement.

### 2.5. Western Blotting Analysis for INSL5 in Human Adipose Tissue

For protein analysis, SAT samples, colon tissue and HeLa cells (as positive controls) were homogenized in RIPA Lysis Buffer with phosphatase and protease inhibitors cocktail. Total protein concentration was determined using a colorimetric assay (Pierce ^TM^BCA Protein Assay kit, #23225, Thermo Fisher Scientific, Waltham, MA, USA). Samples of 30 µg protein were subjected to SDS PAGE using 4-12% polyacrylamide precast gel at a voltage of 160V. Proteins were then transferred to a Hybond ECL nitrocellulose membrane and blocked with 5% (*w*/*v*) dried non-fat milk in Tris buffered saline (TBS)/Tween 20. For INSL5 protein detection, we utilized a 1:1000 diluted rabbit primary antibody (#105325, Abcam, Cambridge, MA, USA), a 1:2500 diluted antibody for leptin (#PA1-052, Invitrogen, Waltham, MA, USA), and a 1:5000 diluted mouse primary antibody for ß-actin (#A5441, Sigma Aldrich, Milan, Italy), used as a reference protein. An IgG-HRP-conjugated secondary antibody was used for protein detection. Immunoreactive bands have been visualized with the ECL plus reagent kit (GE Healthcare Italy, Milan, Italy).

### 2.6. Statistical Analysis

The results were expressed as means ± SD. Data obtained before and after weight loss were compared using the Student’s *t*-test. Correlations were performed by simple linear regression analysis, and Spearman’s correlation coefficients were used to evaluate the correlations between INSL5 plasma levels and other clinical and hormonal parameters. A *p* value < 0.05 was considered statistically significant. The statistical analysis was carried out using GraphPad PRISM software (version 9.5.1; GraphPad Software Inc., San Diego, CA, USA).

## 3. Results

### 3.1. Clinical, Anthropometric, and Metabolic Characteristics of the Obese Patients before and after LSG

Table 1 reports the anthropometric parameters before and one year after LSG in obese patients. As expected, LSG induced a significant body weight reduction along with a significant loss in fat mass and waist circumference. Furthermore, as expected, leptin plasma levels showed a significant reduction one year after LSG, along with fat mass reduction.

### 3.2. Plasma Levels of INSL5 before and after Laparoscopic Sleeve Gastrectomy

**Figure 1:**
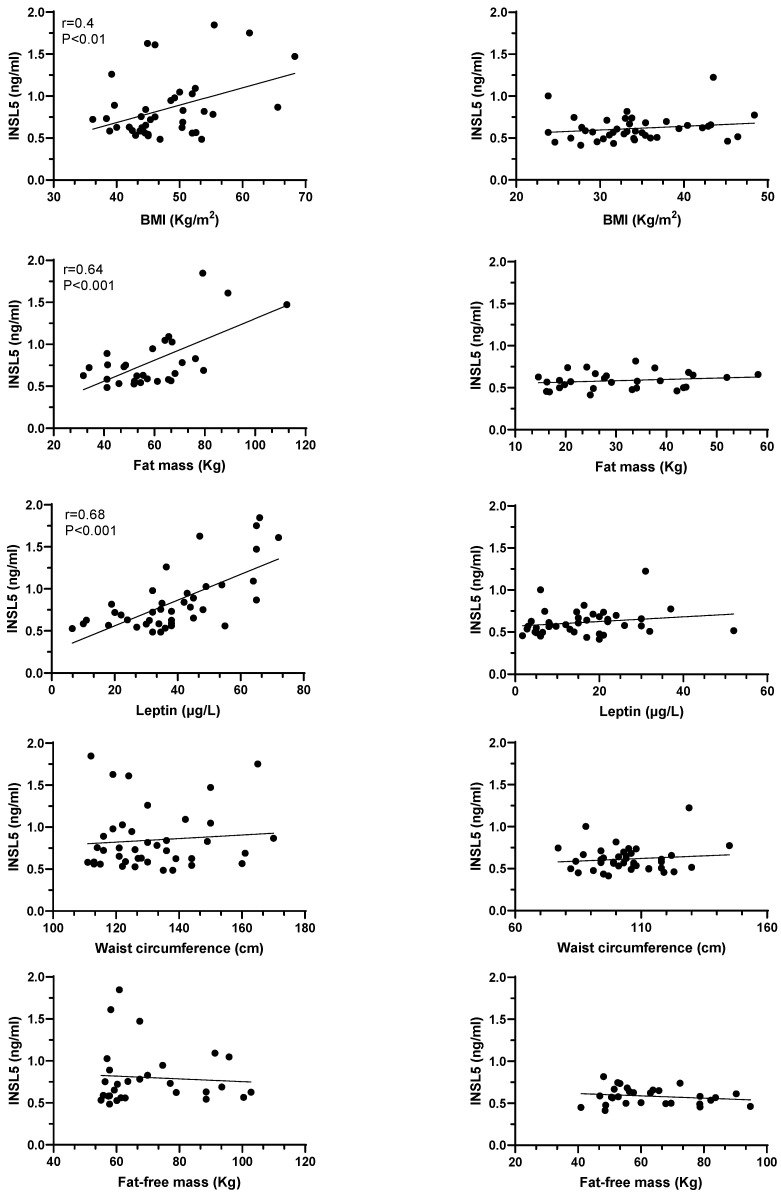
Correlations between INSL5 plasma levels and BMI, fat mass, fat free mass and leptin plasma levels in obese subjects before (left columns) and after (right columns) bariatric surgery. Data for BMI and leptin plasma levels have been obtained in all patients (n = 40). Data for fat mass and fat-free mass have been obtained in 29 patients (see Materials and Methods Section).

**Figure 2:**
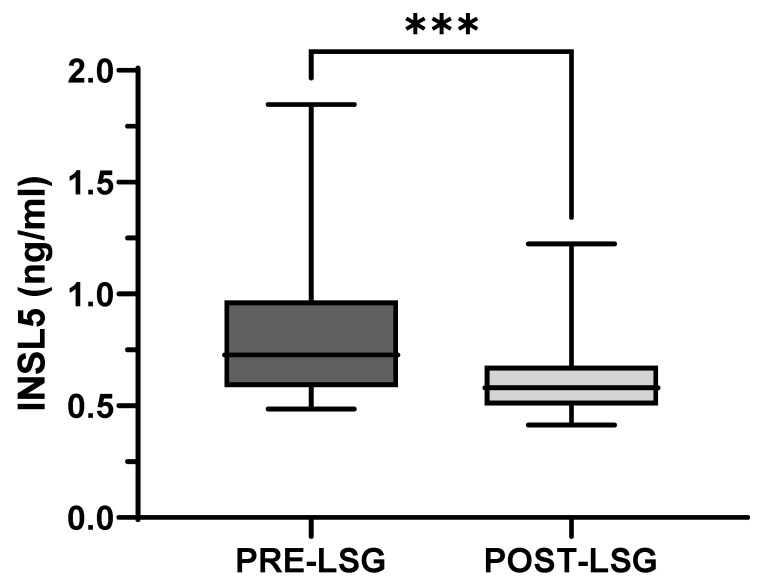
INSL5 plasma levels in obese subjects before (PRE-LSG) and after (POST-LSG) Laparoscopic Sleeve Gastrectomy (LSG). *** *p* < 0.0001.

We did not observe any significant gender difference in plasma INSL5 levels (0.88 ± 0.42 vs. 0.70 ± 0.21, for women and men, respectively, *p* = 0.173), even after adjustment for BMI. Thus, data from men and women were considered as a whole. As shown in Figure 1, INSL5 plasma levels in obese patients before LSG showed a strong positive correlation with BMI and with fat mass. In agreement with these relationships, INSL5 plasma levels were positively and significantly correlated with plasma levels of leptin, the main secretory product of adipose tissue directly related to fat mass. We did not observe any significant correlation between INSL5 plasma levels and both waist circumference and free fat mass (Figure 1). After one year of bariatric surgery and a significant reduction of BMI, waist circumference, and fat mass (see Table 1), we did not observe any significant correlation of these parameters with INSL5 plasma levels (Figure 2).

Interestingly, one year after laparoscopic sleeve gastrectomy, obese subjects showed a markedly reduced body weight and fat mass, together with a significant reduction of INSL5 plasma levels that were 23% lower than those observed before weight loss (Figure 2).

### 3.3. INSL5 mRNA and Protein Expression in Human White Adipose Tissue

**Figure 3:**
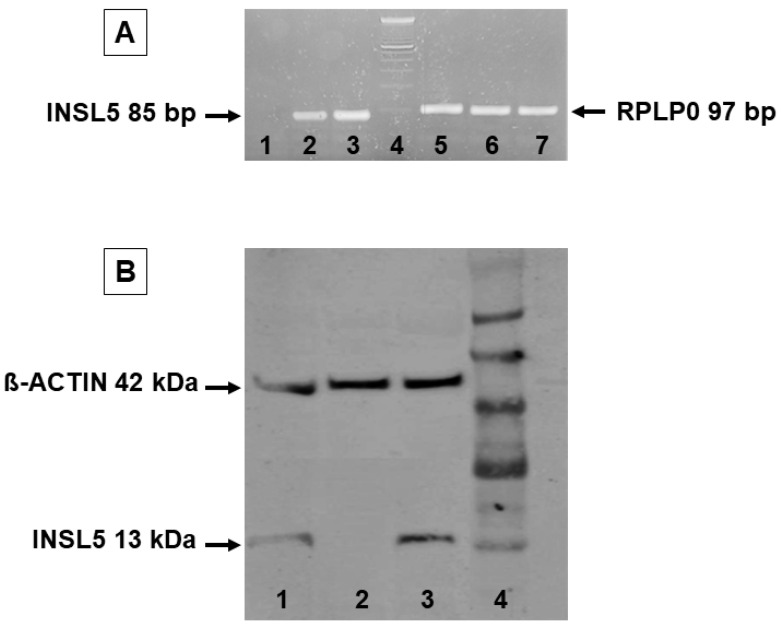
INSL5 is not expressed in human adipose tissue. (**A**): Qualitative RT-PCR analysis of *INSL5* mRNA expression in ex vivo adipose tissue (lane 1), HeLa cells, and colon tissue (positive controls, lane 2 and 3, respectively), 100 bp DNA ladder (lane 4), and RPLP0 housekeeping gene detection (lanes 5–7). (**B**): Western blot analysis of INSL5 protein expression in HeLa cells (lane 1), human subcutaneous adipose tissue (lane 2), and colon tissue (lane 3). Lane 4 reports the Seeblue pre-stained protein standard.

Gene expression data showed the presence of *INSL5* mRNA in the human colon and in HeLa cells used as positive controls, while no amplification plot was obtained from subcutaneous adipose tissue (Figure 3A). The expression of the RpLp0 housekeeping gene was the same in each sample (Figure 3A). The specificity of the real-time PCR for *INSL5* gene expression was determined on an electrophoresis agarose gel, and a single band of 81 bp was obtained only in control samples (Figure 3A).

Similarly, the results from traditional Western blotting provided a main band of 13 KD for INSL5 only in colon tissue and HeLa cells used as positive controls, while no signal was obtained in subcutaneous adipose tissue. The 42 KD b-actin bands were used as a reference control for all samples (Figure 3B).

## 4. Discussion

INSL5 is a novel hormone of the relaxin superfamily that has been first identified as a secretory product of the enteroendocrine L cells of the distal gut (mainly colon and rectum), although it is also expressed in a number of different tissues such as the brain, pituitary, thyroid, kidney, and uterus [16]. In previous animal studies, given its orexigenic properties, INSL5 has been implicated in both mealtime hunger and the regulation of body weight [8,9,10].

Here we show that in humans, the novel orexigenic hormone INSL5 is detectable at significant concentrations in the plasma of obese subjects, with no differences between males and females. In obese patients, we observed that INSL5 plasma levels showed a strong positive correlation between classic markers of obesity such as BMI, fat mass, and leptin plasma levels. Since INSL5 is an orexigenic hormone, positive correlations between INSL5 plasma levels and BMI, fat mass, and leptin levels were expected. These direct and significant correlations were not observed after one year of bariatric surgery and significant reductions in body weight, BMI, fat mass, and leptin plasma levels. Interestingly, after significant weight loss due to LSG, we observed a significant reduction of INSL5 plasma levels. The close direct correlation between obesity markers and INSL5 plasma levels, together with the decrease in INSL5 concentration after fat mass reduction, pointed to adipose tissue as a possible source of INSL5. However, the molecular studies performed in the present study did not show any expression of INSL5 both at the mRNA and protein levels, thus excluding the possibility that the loss of adipose mass after LSG could be responsible for the decrease in INSL5 plasma levels. The lack of any correlation of INSL5 plasma levels with waist circumference would not be surprising since the mechanisms regulating INSL5 secretion are still unclear, and as shown here, the adipose tissue does not seem to have any role in INSL5 secretion. On the other hand, all the studies published so far have pointed to enteroendocrine-L cells as the main (if not unique) source of INSL5 in humans.

The pathophysiological meaning of the present observations is still unclear. It has been recently shown that Insl5^−/−^ mice display alterations in glucose homeostasis and impaired fertility [9]. Furthermore, the expression of INSL5 in the gut of the mouse and the orexigenic effects induced by its administration that are blunted by its blockade suggest that INSL5 might be involved in food intake regulation, as shown for other gut hormones that are secreted by the enteroendocrine cells widely distributed along the gastrointestinal tract. These cells are deputed to sense gut content and to release hormones, such as ghrelin, cholecystokinin, and glucagon, like peptide 1 and peptide YY, that, after entering the circulation or interacting with the gut nervous system, can signal to distant target cells within the brain or act locally on neighboring gut cells and neuronal networks to regulate food intake and thus energy balance and body weight. In this respect, it has been shown that obesity and diabetes mellitus can be associated with alterations of enteroendocrine cell hormonal secretion [8,17]. Indeed, recent studies have demonstrated that only the peripheral and not the intracerebroventricular administration of INSL5 induces an increase in appetite leading to obesity in mice, although data published so far on the expression of the INSL5 receptor RXFP4 in the hypothalamus have shown contrasting results [8,9].

Nevertheless, INSL5 represents the second so far identified gut hormone, after ghrelin, with orexigenic properties [8,9]. In this respect, the reduction of INSL5 plasma levels observed after bariatric surgery-induced weight and fat mass loss could participate in the still unclear mechanisms leading to appetite reduction characterized by these surgical procedures [18]. While it has been recently reported that the signaling pathway activated by INSL5 is activated in target cells expressing its receptor [19,20], the specific signals regulating INSL5 secretion in humans are still unknown. It is possible that energetic substrate intake can regulate its secretion by adipose tissue. In this regard, in mice, INSL5 secretion is reduced by food intake, further underlying the role of this hormone in the regulation of energetic homeostasis. Furthermore, it has been recently reported that in mice, INSL5 influences glucose homeostasis by stimulating insulin secretion from pancreatic islet cells that have been shown to express the INSL5 receptor RXFP4 [9]. Those authors suggested a potential role for INSL5 signaling in the regulation of insulin secretion and pancreatic beta-cell homeostasis [9]. Finally, although there is a strong direct relationship between INSL5 and leptin plasma levels, the role of leptin as a possible mediator of INSL5 secretion needs further study.

## 5. Conclusions

In conclusion, the present study demonstrates that the orexigenic hormone INSL5 is detectable in humans and that its plasma concentrations show a positive correlation with BMI, fat mass, and leptin plasma levels in obese subjects and are significantly reduced after weight loss. These observations could contribute to extending our knowledge of the well-known modulation of the gut orexigenic signals induced after bariatric surgery in obese subjects. Finally, these observations suggest that the modulation of the INSL5/RXFP4 axis might represent a target for the treatment of obesity in humans.

## Figures and Tables

**Table 1:** Body weight parameters and leptin plasma levels before and one year after Laparoscopic Sleeve Gastrectomy (LSG) in obese patients. Data for weight, BMI, waist circumference, and leptin plasma levels have been obtained in all patients (n = 40). Data for fat mass have been obtained in 29 patients (N.A., not applicable).

	Before-LSG	After-LSG	*p*
**Weight** (Kg)	133.2 ± 27.4	95.3 ± 23.1	<0.0001
**BMI** (Kg/m^2^)	47.4 ± 7.0	33.8 ± 6.1	<0.0001
**%Total body weight loss**	-	28.5 ± 8.8	N.A.
**Waist circumference** (cm)	131.4 ± 15.5	104.0 ± 14.6	<0.0001
**Fat mass** (Kg)	59.4 ± 17.5	32.1 ± 13.4	<0.0001
**Leptin** (µg/L)	38.0 ± 16.9	16.1 ± 13.5	<0.0001

## Data Availability

The raw data supporting the conclusions of this article will be made available by the authors upon justified request, without undue reservation.
